# The effects of local rotation on roll vection induced by globally rotating visual inducer

**DOI:** 10.3389/fpsyg.2015.00694

**Published:** 2015-05-27

**Authors:** Shinji Nakamura

**Affiliations:** Division of Clinical Psychology, Faculty of Child Development, Nihon Fukushi University OkudaMihama-cho, Japan

**Keywords:** self-motion, vection, roll motion, roll vection, global motion, local motion

## Abstract

A visual stimulus rotating globally along an observer's line of sight can induce the illusory perception of self-rotation in the opposite direction (roll vection). Psychophysical experiments were conducted to examine the effects of local rotations of visual elements of the stimulus that were manipulated independently of the global rotation. The results indicated that the addition of local rotations inconsistent with the global rotation (assumed to be the primary inducer of roll vection), generally decreased the strength of perceived self-rotation. The uniformity of orientation of the elements composing the global visual pattern and the visual polarities assigned to each visual element, i.e., intrinsic directionality concerning up and down, were observed to function as modulators of the effects of the local rotation. These results suggested that local motion signals arising from independent rotations assigned to each element of a visual object cannot be ignored in the perceptual mechanism underlying roll vection.

## Introduction

Self-motion perception is important for many aspects of our daily activities, especially in the case of locomotion. Without accurate and robust perception of our self-motion, we cannot move around the external environment, avoiding collisions with obstacles. Self-motion perception is mediated by redundant perceptual processing in which multiple sensory organs, including the visual, kinesthetic, somatosensory, or vestibular systems, can contribute. Among these, the visual contribution is dominant (e.g., Howard, 1982). In the case of passive self-motion (e.g., when riding on a cart), the somatosensory and kinesthetic systems cannot inform self-motion correctly. Similarly, the vestibular organ cannot detect self-motion with a constant speed; it can only sense acceleration added to a head. When we move around in a space, visual images of the external scene projected onto our retinae create a global transformation consistent with self-motion; retinal expansion/contraction is obtained by forward/backward self-motion, and horizontal or vertical self-motions yield translational flow on the retinae in the opposite directions (e.g., Andersen, 1986). Retinal image motion of external objects can be caused not only by the motion of these objects, but also by the observer's self-motion through the environment. Uniform retinal image motion across entire area of visual field would more likely be due to self-motion than motion of visual objects. This kind of optic flow should be the sole determiner of our self-motion perception during passive and constant self-motion. The optic flow reflects the speed and direction of self-motion directly, so no accumulation of acceleration or complicated transformation should be needed. It is because of this visual information that we can perceive accurate self-motion under various situations.

The significant contributions of visual information in perceiving self-motion can also be easily understood by a perceptual phenomenon called visually induced self-motion perception, also known as vection (Fischer and Kornmüller, 1930). When we observe a visual stimulus that occupies a larger part of our visual field and moves uniformly, we occasionally perceive that our self/body moves in the opposite direction to that of the visual inducer, although we remain static in reality. Vection shows that visual information can evoke self-motion perception with considerable strength by itself, without accompanying any consistent vestibular or kinesthetic information. The relationship between visual input and resultant perceptual output in an experimental vection situation is exactly the same as in the case of real self-motion experienced in the natural environment. Thus, researchers who are interested in the perceptual system responsible for self-motion perception often investigate vection in order to examine the effects of visual stimulation. They have tried to reveal which factors in the visual stimulus can affect and modulate the occurrence and strength of vection, and have found many important facts that are not only helpful in understanding the natures of our perceptual processing concerning self-motion but also beneficial in developing an effective self-motion display that can be utilized in virtual reality systems (see Riecke, 2010 for a review).

Natural self-motions generate global transformations of retinal image of the static external world, and thus, we can rely on it in perceiving self-motion. Of course, the global motion is not a sole determiner of self-motion perception; there are a number of visual factors which can affect occurrence and strength of visual self-motion (see Warren, 1995 and Riecke, 2010 for reviews concerning various factors that might affect vection). Indeed, visually evoked postural response, which is supposed to share common underlying mechanism with vection but rather be controlled by automatic and subconscious systems, was affected not only by simple global motion of the visual inducer but also by other factors, including three dimensional geometries of the visual inducer or interpretation of the environment (e.g., Guerraz et al., 2001; Meyer et al., 2013). Spontaneous postural response is still affected by factors other than the global motion, and should be much more in vection which can be considered to involve rather cognitive and conscious processes.

Nevertheless, a global transformation of the retinal image would be primary factor in visual self-motion perception (e.g., Andersen, 1986). The local motion of each element in the visual pattern should be integrated across a wider area of the visual field before self-motion is calculated via perceptual processing. As described earlier, self-motion perception is critically important in our behavior. Thus, global motion integration, thought to be the basis for visual self-motion perception, is implemented by our visual system in a robust (noise-resistant) manner; we can extract the global motion component even in conditions where only a small number of visual elements (e.g., less than 5%) are set to move coherently against the other noise elements that move in random directions (e.g., Pilly and Seitz, 2009). Indeed, vection can also be induced in situations where the motion coherency of elements in the visual pattern is detracted, although vection strength surely becomes weaker than it would be in a case with fully coherent conditions (e.g., Nakamura, 2010; Saito and Sakurai, 2014).

While the global integration of visual motion seems to be important in understanding the perceptual mechanism underlying self-motion, vection research has rarely addressed the interactions between local and global motions. Therefore, in this study, I introduce local motion of visual components manipulated independently of the global motion of a pattern as a modulator of self-motion perception, which might be primary generated by the global motion, as a first step toward fully understanding local-global interactions in perceiving self-motion. In the psychophysical experiments reported in this article, the visually induced perception of self-rotation around the observer's line of sight, namely roll vection, was investigated under various stimulus conditions. A visual stimulus with continuous rotation around the center of the visual display can induce an illusory perception of self-rotation along the roll axis toward the opposite direction (e.g., Dichgans et al., 1972; Held et al., 1975). Employing visual rotation with a roll axis enables us to set each visual component to locally rotate on the spot, avoiding variations in its global position, and thus, global motion will not be affected by the local manipulations. We can manipulate local and global rotations in a completely independent manner in the case of roll vection. This would be difficult to achieve using visual expansion/contraction that induces self-motion in depth or visual translations resulting in horizontal or vertical self-motions (Please refer Supplemental Materials for examples of visual stimulus employed in psychophysical experiments reported in this article).

In Experiment 1, an eight-pointed spoke-like star made from a combination of four short line segments was employed as a visual object that composed the stimulus pattern. The visual pattern was set to rotate globally around the center of the visual display, while each of the star-shaped elements was also manipulated to rotate on the spot around its center, independently of the global rotation. This stimulus design enabled us to investigate the effects of the local rotation on roll vection independently of the global rotation. Experiment 2 replicated Experiment 1, but with the visual star-shape objects being replaced by multiple copies of the same human face. Previous studies investigating roll vection have revealed the significant impact of visual polarity contained in the visual object (e.g., Howard and Childerson, 1994; Howard and Childerson, Allison et al., 1999; See also Palmisano et al., 2006). They maintain that certain visual objects such as people, plants, or furniture are always associated with an intrinsic visual polarity because they have identifiable orientations (in terms of what is “up” and “down”) in accordance with the context of the natural scene, and may thus have facilitative effects on roll vection. The polarities of the visual elements would be especially important when considering the effects of local rotation; hence, a human face—as one of the instances of visual objects holding strictly defined visual polarities—was employed as an object of the stimulus pattern.

## Materials and methods

### Ethics statement

The experiments reported here were checked and approved in advance by the Ethical Committee at Nihon Fukushi University. Written informed consent was obtained from each participant.

### Participants

Twenty-one undergraduate students (7 males and 14 females, age range: 19–25 years) volunteered to participate in both in Experiments 1 and 2 as observers. All participants had normal or corrected-to-normal visual acuities, with no self-reported vestibular impairments. Though some of them had previous experiences of participating in vection experiments, none of them were aware of the purpose of the experiments.

### Apparatus

The visual stimulus was presented on a 55-inch flat-screen LCD display whose size was 64 cm in height and 120 cm in width (Hisense HS55K20). The spatial resolution of the visual display was 1080 pixels in height and 1920 pixels in width, and the refresh rate was 60 Hz. The participants observed the visual display through a rectangular viewing hole, which limited their field of view so that no visual objects other than the stimulus were observable. They observed the stimulus at a viewing distance of 86 cm, and the visual display subtended 43 vertical and 90 horizontal degrees in the visual angle. The experimental trials were carried out in a completely dark experimental room; the visual display was the sole light source in the room. A personal computer with an OpenGL-compatible graphics card was used for presentation of the visual stimulus. An additional mouse connected to the stimulus-generating PC was utilized to measure the participant's response.

### Stimulus

The visual stimulus rotating around the center of the visual display was employed to induce the observer's roll vection. The rotation speed was fixed to 60°/s, which was confirmed as approximately optimal by our previous informal observations. Previous studies revealed that there would be no effects of rotation direction on vection (e.g., Allison et al., 1999), and thus, the direction of the rotation (clockwise or counterclockwise) was randomly determined in each experimental trial. However, it might be helpful to avoid the accumulation of adaptation to unidirectional rotation. A red fixation cross whose size was 2° in the visual angle was always present at the center of the display during the experimental trials.

**Figure 1:**
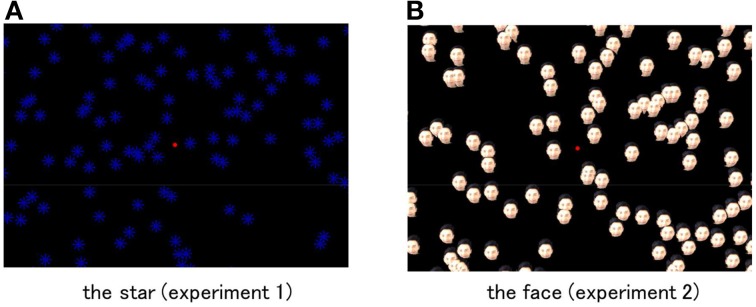
**Visual stimuli employed in Experiment 1 (A: the star) and 2 (B: the face)**. Stimulus dimensions were modified from the real stimulus because of visibility. The red spot in the figure indicates the location where the fixation cross was presented in the visual stimulus employed in the experiment.

The visual elements composing the global stimulus patterns were “eight pointed spoke-like stars (star)” in Experiment 1 and “face of a Japanese male (face)” in Experiment 2. The star was employed as an example of a visual object without intrinsic polarity (i.e., an identifiable top and bottom), and the face was selected as an instance of a visual object with a strong visual polarity. The star consisted of four short line segments, whose length was 4° and width was 0.2°, crossing each other at their centers with 45° angles. The color of the star was blue with a luminance of 15.7 cd/m^2^. The face was accomplished by mapping a full color image of the face of an ordinary Japanese man onto the circular shaped stimulus. The face element subtended 4° in the visual angle, which was the same as in the case of the star-shaped element. The average luminance within the face stimulus was 17.2 cd/m^2^, which was also approximately the same as that of the star. The background of the visual display was black (0.7 cd/m^2^). All of the visual elements, those of the star tested in Experiment 1 and the circular ones of the face tested in Experiment 2, were manipulated to rotate locally at their position around the center, independently of the global pattern rotation. On an average, 250 visual elements, making up either the star or the face, were randomly positioned in the visual stimulus. Figure 1 illustrates visual stimuli employed in Experiments 1 and 2.

There were two independent variables in either experiment. The first one was the initial orientation of the visual elements. In *the uniform initial orientation condition*, all visual elements were placed correctly upright at the beginning of each stimulus presentation. In the star stimulus (Experiment 1), one of the four line segments was set to be vertical, and another one was set to be horizontal. In the face stimulus (Experiment 2), the face image was presented in a naturally upright orientation. During the stimulus rotation, each element's orientation was kept consistent except for the random rotation condition described later. In *the random initial orientation condition*, the initial directions were randomized from 0 to 360° for each of the visual elements.

The second independent variable was the local rotation of the visual elements, and there were five different styles of rotation. In the *no local rotation condition*, each visual element rotated globally but did not change its orientation. Thus, there was no local visual rotation in this condition. Note that this might be equivalent to the retinal motion in the situation where the observer rotates spatially (the observer's self-rotation causes global rotation of the visual pattern) while each visual element rotates on the spot in the same direction and at the same speed as the observer's rotation. In the uniform initial orientation condition, the visual element was kept in an upright position throughout the duration of stimulation, with no local rotation. In the *consistent local rotation condition*, each visual element changed its orientation consistently with the global rotation. This would be equivalent to the observer's self-rotation in the case where the visual pattern and the element contained in it were static in the external environment. In the *same direction* and *opposite direction local rotation conditions*, visual elements rotated locally in the same or opposite direction as the global rotations, respectively. The former situation could be obtained when the visual objects externally rotate at the spot in the opposite direction as the observer's self-rotation with the same speed, and the latter case might reflect local rotation of the visual objects in the same direction but at twice the speed of the observer's self-rotation. In the *random local rotation condition*, the speed and direction of the local rotation assigned for each visual element were randomly determined, with ranges the same as those in the same direction and opposite direction conditions. The random local rotation eliminated the difference between the uniform and random initial orientation conditions during the stimulus rotation; the difference between these two conditions was only observable at the beginning of the stimulus presentation (before the rotation started). Except for in the consistent rotation condition, all the local rotations might cause inconsistent local rotations against the global rotation; the visual elements should rotate on the spot independently of the observer's rotation assumed to be defined by the global rotation. Sample movies of visual stimulus employed in the experiments were provided as Supplemental Materials (Movies 1, 2, and 3 correspond to the no, consistent, and random local rotation conditions in Experiment 1, and Movies 4 and 5 correspond to the same directional and no local rotation conditions, respectively as examples). It should be noted that, in demo movies, stimulus dimensions (e.g., stimulus sizes and densities) were modified from the real stimulus because of visibility.

### Procedure

The participants sat on a chair in front of the visual display and observed the visual stimulus presented on it through the viewing hole. Due to the viewing hole, the observer's head was practically kept spatially immovable. The participant's task was to report perceived self-rotation (roll vection) by pressing a mouse button. It was emphasized that he or she needed to keep pressing the button as the roll vection continued, release it as soon as roll vection disappeared, and press it once again when roll vection came again. After each stimulus observation, which lasted 30 s, the participants were required to estimate the strength of the roll vection experienced during the trial, using roll vection induced by the standard stimulus as a modulus. The standard stimulus was approximately identical to the one used in the experimental conditions, but the visual elements were blue circles whose radius was 4°. The participants were instructed to estimate the strength on a scale from 0 for “no roll vection at all” to 50 for “roll vection was as strong as with the standard stimulus,” or beyond. In order to establish the standard of the strength estimation and allow the participants to become familiar with the experimental procedure, observations of the standard stimulus were repeated four times before the experimental trials as a demonstration.

Experiments 1 and 2 were executed on different days. There were 10 stimulus conditions, i.e., two initial orientation conditions (uniform and random) × five local rotation conditions (none, consistent, same direction, opposite direction, and random). The trials for each condition were randomly repeated four times, and thus, each participant executed 40 trials in each of Experiments 1 and 2.

At the beginning of the stimulus presentation, a static version of the visual stimulus was presented for 3 s, followed by 30 s of stimulus rotation, and then the stimulus disappeared. A white noise pattern was presented for 2 s after the rotating stimulation was terminated, to prevent visual adaptation. Including the time needed for strength estimation, an interval longer than 30 s was ensured between each trial. After each of the 10 trials, a 5-min rest period was inserted. The participants could request to observe the standard stimulus again after the rest. To accomplish all the experimental trials, approximately 90 min were required for each participant, including instructions and training trials.

## Results

### Data analysis

Onset latency and accumulated duration were calculated based on the participant's button pressing responses. There were three different vection indices, namely, latency, duration, and estimated strength. Stronger vection tends to have shorter latency, longer duration, and higher strength estimates. In most trials, the participants experienced roll vection in the direction opposite to the global rotation of the stimulus pattern, while its latency, duration, and strength estimate varied depending on the stimulus conditions. In exceptional cases where no roll vection was reported, latency was assigned 30 s (the same as the stimulus duration), and duration and estimation were 0. There were no trials where the participant reported that his or her body rotated in the same direction as the global stimulus rotation. A repeated-measurement analysis of variance (ANOVA) with a factorial design of 2 (initial orientation) × 5 (local rotation) and *post-hoc* multiple comparisons were applied for each vection index. Minimum significance level was set to 5%.

### Experiment 1

**Figure 2:**
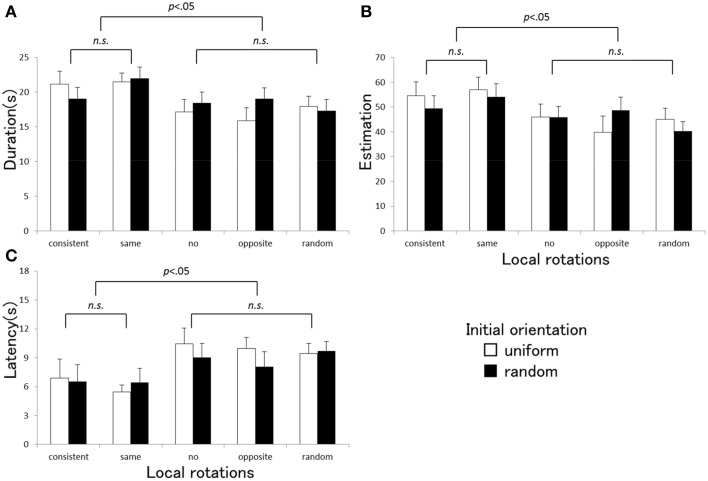
**Averaged duration (A), estimation (B), and latency (C) obtained under each stimulus condition in Experiment 1**. Error bars indicate SEMs. *P*-values indicate results of multiple comparisons between the local rotation conditions.

The ANOVA indicated that there was a significant main effect of the local rotation of the visual elements for each vection index [duration: *F*_(4, 80)_ = 6.06, *p* < 0.001, η^2^_*p*_ = 0.23, estimation: *F*_(4, 80)_ = 4.83, *p* = 0.002, η^2^_*p*_ = 0.20, latency: *F*_(4, 80)_ = 8.92, *p* < 0.001, η^2^_*p*_ = 0.65]. The main effect of the initial orientation was not significant [duration: *F*_(1, 20)_ = 0.61, *p* = 0.44, η^2^_*p*_ = 0.03, estimation: *F*_(1, 20)_ = 0.24, *p* = 0.63, η^2^_*p*_ = 0.01, latency: *F*_(1, 20)_ = 79, *p* = 0.39, η ^2^_*p*_ = 0.04]. Interaction between the local rotation and the initial orientation was significant for duration and estimation [duration: *F*_(4, 80)_ = 3.01, *p* = 0.023, η^2^_*p*_ = 0.13, estimation: *F*_(4, 80)_ = 2.90, *p* = 0.027, η ^2^_*p*_ = 0.13], but not significant for latency [*F*_(4, 80)_ = 0.83, *p* = 0.51, η ^2^_*p*_ = 0.02]. The significant interaction confirmed in duration and estimation might reflect the tendency that the effects of the local rotation were amplified under the uniform initial orientation condition more than in the random condition. Multiple comparisons using Tukey's HSD test revealed that there were no significant differences among the three vection indices between the consistent and the same directional local rotation conditions, and among the no, opposite, and random rotation conditions. A statistical significance was only found between these two subgroups. Figure 2 presents the average vection indices (A: duration, B: estimation, C: latency) measured under each stimulus condition in Experiment 1. Stronger vection with shorter latency, longer duration, and higher strength estimate tended to be induced in the consistent and the same direction local rotation conditions more than in the other three conditions with no, opposite direction, or random rotations.

### Experiment 2

**Figure 3:**
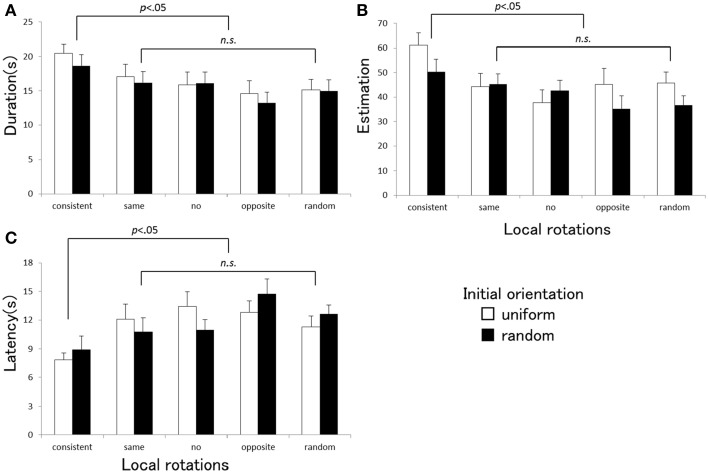
**Averaged duration (A), estimation (B), and latency (C) obtained under each stimulus condition in Experiment 2**. Error bars indicate SEMs. *P*-values indicate results of multiple comparisons between the local rotation conditions.

The ANOVA indicated that a main effect of the local rotation was significant for each vection strength index [duration: *F*_(4, 80)_ = 3.67, *p* = 0.009, η^2^_*p*_ = 0.16, estimation: *F*_(4, 80)_ = 3.47, *p* = 0.012, η^2^_*p*_ = 0.15, latency: *F*_(4, 80)_ = 8.92, *p* = 0.001, η^2^_*p*_ = 0.21]. The main effect of the initial orientation was significant only in estimation [*F*_(1, 20)_ = 6.74, *p* = 0.017, η^2^_*p*_ = 0.25], but not in duration or latency [duration: *F*_(1, 20)_ = 0.70, *p* = 0.41, η^2^_*p*_ = 0.03, latency: *F*_(1, 20)_ = 0.067, *p* = 0.80, η^2^_*p*_ = 0.003]. No significant interactions between the two factors were found [duration: *F*_(4, 80)_ = 0.087, *p* = 0.98, η^2^_*p*_ = 0.004, estimation: *F*_(4, 80)_ = 1.53, *p* = 0.20, η^2^_*p*_ = 0.071, latency: *F*_(4, 80)_ = 1.20, *p* = 0.32, η^2^_*p*_ = 0.057]. Multiple comparisons showed that three vection indices obtained in the consistent local rotation condition were significantly different from those of the other four conditions (none, same, and opposite directions and random rotations). Figure 3 shows the averaged vection indices (A: duration, B: estimation, C: latency) measured under each stimulus condition in Experiment 2. Vection was stronger in the consistent local rotation condition than in the other local rotation conditions, with shorter latency, longer duration, and higher strength estimates. The vection obtained under the uniform initial orientation condition was generally estimated as stronger than the one under the randomized orientation condition.

## Discussion

### The effects of local rotation of the visual elements

The current study tried to investigate the effects of local motion of the visual objects composing a global visual pattern on human self-motion perception, by analyzing the roll vection induced by global visual rotations. Using roll vection and rotating visual stimuli, we can create a situation where the visual elements can rotate on the spot, without distracting the global transformation of the visual pattern. As the visual elements, Experiment 1 employed an eight-pointed star, while a human face was used in Experiment 2 because it can be considered highly polarized. The results of the two psychophysical experiments revealed that roll vection became stronger in the condition where the rotations of the visual elements were consistent with the global rotation of the visual stimulus. The visual stimulus presented in the consistent local rotation condition would be equivalent to the observer's self-rotation in the case where the visual pattern and the element contained in it were static in the external environment. The results of the experiments succeeded in adding new evidence for a fact that has been established by many vection studies over the years, that is, visual motion consistent with the observer's self-motion in a static visual environment can induce strong vection (e.g., Howard, 1982). It should be worthy to note that Palmisano et al. (2006) revealed that compelling roll vection could still be induced under conditions where visual scene was perceived to be sheared and distorted. Shearing transformation of the visual scene would cause another type of local motion signals which were inconsistent with the global rotation, and thus, the finding by Palmisano and his colleagues might be inconsistent with the present experiments which indicated that inconsistent local rotation inhibited roll vection. On the other hand, Nakamura (2010) showed that tensile and compression of visual stimulus strongly detracted translational self-motion perception (linear vection). We need further experiments to examine the effects of local motion on vection, especially in order to shed a light on singularity of roll vection.

In the conditions where the visual elements rotated inconsistently with the global pattern rotation (none, same, and opposite directions and random local rotations), vection strengths were decreased, as exhibited by the longer latency, shorter duration, and lower estimation. Thus, inconsistent local rotation could act as an inhibitor of self-motion perception, even in situations where the local motion of the visual elements never disturbs the global rotation in the entire visual pattern. The present investigation, together with a huge accumulation of previous research concerning visually induced self-motion perception and visually evoked postural responses, indicates that perceptual process responsible for perception and control of self-motion can not be determined merely by the output of the global motion detector, which supposedly exists in a specific brain area (likely dorsal medial superior temporal; MSTd) involved in human visual pathway (e.g., Saito et al., 1986; Duffy and Wurtz, 1991; see also Palmisano et al., 2015). The local motion signal is clearly not negligible in vection even if it is irrelevant to global motion integration. Strength estimates in most of the stimulus conditions with inconsistent local rotations were significantly lower than 50; vection was weaker in those conditions than when induced by the standard stimulus. The standard stimulus consisted of the visual elements with simple circle shapes. Thus, with the standard stimulus, local rotation could not be extracted, and instead, only global rotation existed. This result clearly indicated that the inconsistent local rotations generally inhibited roll vection induced by the global rotation of the visual pattern.

An exception existed in Experiment 1; in the condition with the same directional rotation, vection strength was as strong as the one with consistent rotation, indicating no decrement caused by the inconsistent local rotation. One possible explanation would be as follows: local rotation of an individual visual element that is inconsistent with, but in the same direction as the global rotation tends to elicit opposite self-rotation by itself (just like the regular relationship between self-motion perception and visual inducer's motion), and this self-motion component compensates for the decrement of globally induced roll vection caused by the inconsistency between the local and global rotations. In Experiment 2, which employed a face as the visual element, vection strength in the same directional rotation condition was significantly lowered from that of the consistent condition, similar to the other inconsistent conditions. It can be assumed that, with highly polarized visual elements, i.e., the face, the effects of inconsistent local rotations would be much more amplified than when using less polarized elements, such as the star in Experiment 1, and thus, the decrement of vection caused by local-global inconsistencies cannot be fully compensated for by the additional facilitative effect of the same directional local rotation.

### The effects of consistency in the visual orientations

The experiments reported here manipulated the initial orientations of the visual elements to create uniform and random initial orientation conditions. In the uniform condition, all elements rotated with consistent orientations throughout the stimulus duration, except for in the random local rotation condition. In Experiment 1, the interaction between the initial orientation and the local rotation was significant for duration and estimation; the effects of the local rotation became stronger with the uniform visual orientations assigned to each visual element. It might be plausible to consider that the uniformly aligned orientations of the visual elements amplified the effects of local rotations of the visual objects and intensified the inhibition of roll vection caused by the inconsistent local rotation.

In Experiment 2, in which the visual stimulus consisted of strongly polarized visual elements (i.e., the face), a significant main effect of the initial orientation was confirmed for estimation; vection induced by the visual stimulus with the uniformly orientated elements tended to be estimated stronger than the one with randomized orientations. The previous studies investigating roll vection have suggested that a stimulus that contains many highly polarized (and consistently oriented) visual objects can induce stronger vection (e.g., Howard and Childerson, 1994; Allison et al., 1999). Randomized orientation assigned to the visual elements would destroy the advantage of the visual polarities. This might be supported by the fact that the significant facilitation of vection from baseline strength with the standard stimulus (estimation value of 50) caused by the consistent local rotation was only confirmed in the uniform initial orientation condition in Experiment 2, but not in the randomized orientation condition, nor in Experiment 1 where the elements with less visual polarity were employed. In Experiment 2, there was no significant interaction between the local rotation and initial orientation. One explanation for this result is that the effects of local rotation might be so intensified that decrement caused by the randomized orientation would hardly be confirmed in the condition where the visual elements contained high spatial polarities.

### Possible effects of motion adaptation and perceived speed of global rotation

Previous vection studies have indicated that certain manipulations of the visual stimulus can affect the strength of self-motion perception via modulation of the observer's adaptation toward the inducer's motion (e.g., Seno et al., 2010, 2011; Kim and Khuu, 2014). Similarly, other studies have pointed out that there are visual factors that have some impacts on vection by affecting the perceived speed of the visual inducer (e.g., Graaf et al., 1991). Thus, additional control experiments were carried out in order to confirm the possible effects of modulations in the motion adaptation and the perceived speed, employing the same apparatus, stimulus, conditions, and participants as Experiment 1. In the motion-adaptation experiment, the participant observed the rotation of the visual pattern for 30 s, maintaining the mouse-button press. After the duration of the motion adaptation, the visual pattern was stopped, but the static pattern remained present on the visual display. The participant was instructed to release the mouse button as soon as the motion aftereffect (perceived rotation of the stimulus pattern in the opposite direction to the global stimulus rotation) disappeared. By measuring the duration of the motion aftereffect, we can obtain the degree of motion adaptation caused by the prolonged exposure to the global rotation under each stimulus condition. In the perceived speed experiment, the participant observed the rotating pattern and estimated speed of the global rotation of the stimulus after each stimulus observation lasted for 30 s. The same standard stimulus (a visual pattern that consisted of simple blue circles) was employed as a modulus, and the perceived speed of the rotation of the standard stimulus was assigned to 50.

Table 1 shows the average duration of the motion aftereffect measured under each stimulus condition. There were no significant main effects of the local rotation [*F*_(4, 80)_ = 0.14, *p* = 0.97, η^2^_*p*_ = 0.007] or initial direction [*F*_(1, 20)_ = 3.65, *p* = 0.07, η^2^_*p*_ = 0.015], nor was there interaction between them [*F*_(4, 80)_ = 0.20, *p* = 0.94, η^2^_*p*_ = 0.010]. The durations of the motion aftereffect described in Table 1, ranging around 2 s, prove *post-hoc* that the inter-trial intervals secured in the main experiments (30 s) were enough long to escape from the artifacts of the motion adaptation. The perceived speed of the stimulus pattern listed in Table 2 also did not exhibit statistical significances in either main effects [local rotation: *F*_(4, 80)_ = 1.36, *p* = 0.25, η^2^_*p*_ = 0.064, initial orientation *F*_(1, 20)_ = 1.15, *p* = 0.30, η^2^_*p*_ = 0.050] or interaction [*F*_(4, 80)_ = 0.54, *p* = 0.70, η^2^_*p*_ = 0.026]. Table 3 summarizes the Pearson's coefficients of correlation between the duration of motion aftereffect and estimated speed of the stimulus rotation against three vection strength indices, i.e., duration, strength estimates, and latency. Neither motion aftereffect nor perceived speed showed significant correlations with vection strength. Thus, the supplemental experiments reported in this section suggested that the effects of the local rotation on vection confirmed by the main experiments were not due to the modulations of the motion adaptation or perceived speed of the global rotation; signals from local rotation detectors may affect perceptual mechanism underlying roll vection in rather direct ways.

**Table 1:** **Duration of motion aftereffect measured under each stimulus condition (seconds)**.

**Initial orientation**	**Local rotation**				
	**No**	**Consistent**	**Same**	**Opposite**	**Random**
Uniform	1.94(0.18)	2.03(0.16)	2.09(0.24)	2.08(0.22)	2.01(0.26)
Random	1.95(0.17)	1.82(0.26)	1.96(0.17)	1.86(0.17)	1.87(0.16)

*Values in parentheses indicate SEMs*.

**Table 2:** **Estimated speed of the stimulus rotation under each stimulus condition**.

**Initial orientation**	**Local rotation**				
	**No**	**Consistent**	**Same**	**Opposite**	**Random**
Uniform	50.47(1.28)	52.142(1.26)	51.23(1.11)	50.52(1.21)	48.19(1.43)
Random	48.90(1.07)	48.19(1.16)	46.52(0.97)	50.57(1.15)	48.047(1.09)

*Values in parentheses indicate SEMs*.

**Table 3:** **Pearson's coefficients of correlation between duration of motion aftereffect and estimated speed of the stimulus rotation against three vection indices**.

	**Vection strength index**		
	**Duration**	**Estimation**	**Latency**
Motion after effect	0.11	0.03	−0.07
Estimated speed	−0.10	−0.07	0.08

### The effects of the stimulus element (star vs. face)

**Figure 4:**
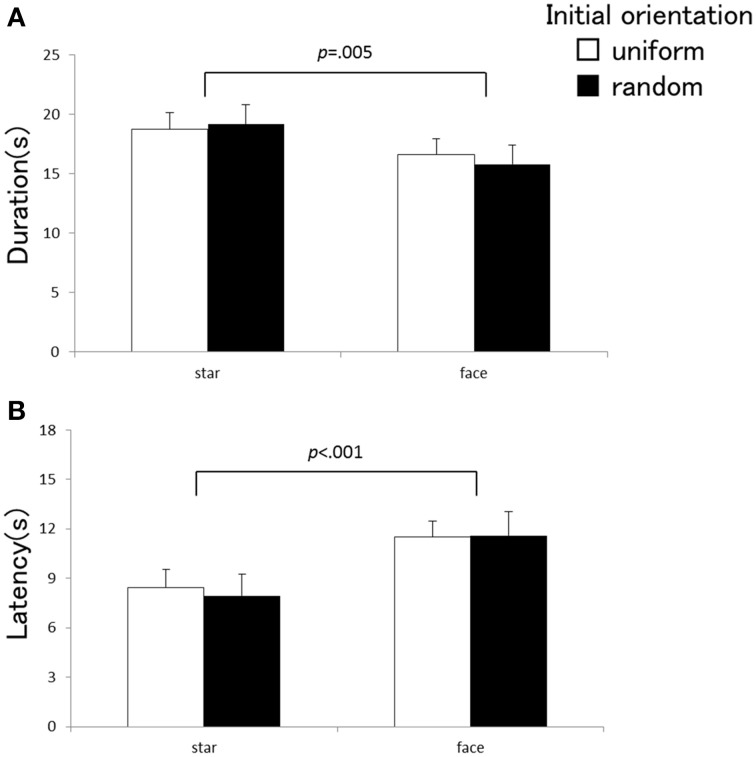
**Averaged duration (A) and latency (B) as a function of the stimulus element (star vs. face)**. Each index was averaged across local rotation conditions. Error bars indicate SEMs.

Experiments 1 and 2 employed identical apparatuses, experimental design, and observers, with a sole difference of the types of the visual element composing the global visual pattern. The star used in Experiment 1 contained less visual polarity than the face used in Experiment 2. The face can be considered richly polarized in a vertical direction because it has a natural orientation as to what is “up” and “down.” We can examine the effects of the visual elements by comparing vection strengths between Experiments 1 and 2. A Three-Way repeated measurement ANOVA with a factorial design of 2 (types of the visual elements) × 2 (initial directions) × 5 (local rotations) was carried out for each of the three vection indices. The main effect of the element types was significant for the vection time course indices [duration: *F*_(1, 20)_ = 9.68, *p* = 0.005, η^2^_*p*_ = 0.33, latency: *F*_(1, 20)_ = 20.60, *p* < 0.001, η^2^_*p*_ = 0.51], but not for estimation [*F*_(1, 20)_ = 1.49, *p* = 0.24, η^2^_*p*_ = 0.07]. The elements types did not exhibit significant interactions against the local rotations [duration: *F*_(4, 80)_ = 1.56, *p* = 0.20, η^2^_*p*_ = 0.072, estimation: *F*_(4, 80)_ = 2.03, *p* = 0.098, η^2^_*p*_ = 0.092, latency: *F*_(4, 80)_ = 2.20, *p* = 0.077, η^2^_*p*_ = 0.099] or the initial orientations [duration: *F*_(1, 20)_ = 2.26, *p* = 0.15, η^2^_*p*_ = 0.10, estimation: *F*_(1, 20)_ = 2.40, *p* = 0.13, η^2^_*p*_ = 0.11, latency: *F*_(1, 20)_ = 0.77, *p* = 0.39, η^2^_*p*_ = 0.37]; three-way interaction was not significant either [duration: *F*_(4, 80)_ = 1.02, *p* = 0.40, η^2^_*p*_ = 0.048, estimation: *F*_(4, 80)_ = 2.20, *p* = 0.076, η^2^_*p*_ = 0.099, latency: *F*_(4, 80)_ = 1.63, *p* = 0.18, η^2^_*p*_ = 0.075]. Figure 4 indicates the latency and duration of vection averaged across the local rotations for the two types of visual elements. Vection occurred later and became shorter with the visual stimulus that consisted of the face (Experiment 2) in comparison with the pattern composed by the star (Experiment 1).

Previous investigations have indicated that the inclusion of richly polarized visual objects in visual inducer was one of the key factors in inducing strong roll vection (e.g., Howard and Childerson, 1994; Allison et al., 1999). The current results showing that a visual stimulus with more polarized elements induced weaker vection seem to be inconsistent with the previous findings. In the current visual stimulus, many isolated human faces were randomly placed in the visual display and, in some conditions, rotated inconsistently against the global rotation. This situation was definitively less natural and lacked ecological validity. Many psychophysical studies have pointed out that the ecologically valid visual inducer, i.e., visual stimulus which can be considered as a natural visual environment surrounding the observers and being static externally, would be highly important in inducing stronger self-motion perception (e.g., Bonato and Bubka, 2006; Bubka and Bonato, 2010). With less ecological validity, such as in the case of Experiment 2, the visual polarities would not exert facilitative effects on roll vection. Indeed, with uniform orientation and consistent local rotation, which can moderate the invalidities, the visual pattern with the face could induce vection with a significantly higher strength estimate than the standard stimulus (see Figure 3). Furthermore, the human face might be one of the most important visual stimuli for human observers, and thus, resources in visual processing would be largely assigned for handling the meaning of the visual objects at the initial period of the stimulus presentation, when the human face was employed as a component. This might be one of the reasons that vection induced by the visual stimulus with the face occurred later and was shorter. Nevertheless, the effects of the polarities contained in the visual stimulus must be investigated once again under a natural visual situation^1^.

### Consistency among the indices of vection strength

The present experiments employed three separate indices for measuring the strength of vection induced under each stimulus condition, namely, onset latency, accumulated duration, and estimated strength. These indices were commonly used in many vection studies and demonstrated that they were highly consistent and in harmony with each other. They were assumed to reflect the same phenomenological aspects of self-motion perception; stronger vection tends to have a shorter latency, longer duration, and higher strength estimate. This was also the case in the present experiments. Table 4 indicates the coefficients of correlation among three vection indices obtained in Experiments 1 and 2. The correlations were considerably high and significant for all combinations of the indices. On the other hand, in terms of the statistical significance of the effects of the independent variables, some inconsistencies were found among the vection indices in the current experiments. For example, in Experiment 1, the interaction between the local rotation and initial orientations was significant only for duration and estimation, but not for latency. In Experiment 2, the main effect of the initial orientation of the visual elements was only significant for estimation, while no significant effects were confirmed for duration and latency. It has been known that roll vection is more unstable in its nature as compared with yaw or linear vection (e.g., Tanahashi et al., 2012); roll vection can often be subject to sudden disruptions, or even directional reversals (though the latter was not confirmed in the current experiments). The present study also found that vection onset latencies were quite long under some conditions (around 10 s). These values were considerably longer than in the case of other types of vection (see Warren, 1995 as a review). In such situations, time course data measured as indices of vection strength (latency and duration) might be somewhat erroneous, and researchers who want to measure the strength of roll vection accurately are encouraged to include strength estimation as one of the measurements.

**Table 4:** **Peason's coefficients of correlation among three vection indices**.

	**Estimation**	**Latency**
Duration	0.73	−0.78
Estimation		−0.62

## Conclusions and future work

The current investigation examined the effects of consistent and inconsistent local rotations on roll vection induced by a global rotation of the visual pattern. The results showed that the effects were exerted mainly in a suppressive manner; the strength of roll vection became generally weaker with inconsistent local rotations. The effects of the local rotations were intensified with uniform orientations of the elements in the condition where less polarized visual objects were employed, while uniform orientations facilitated roll vection in the case of highly polarized visual objects. The present experiments suggested that roll vection is not determined only by the output of a global rotation detector, but also affected by the motion signals arising from local rotation, not via avoiding motion adaptation or modulating the perceived speed of the global rotation. Future experiments are still needed for a better understanding of the perceptual system responsible for roll vection. For example, the effects of the visual polarities should be examined using a stimulus situation that can be considered as more natural and ecologically valid. Moreover, in the present experiments, the participants viewed the visual stimulus through a rectangular viewing hole, so there was always a static visual frame. The visual frame can be one of the predominant factors in an observer's self-orientation with a roll axis, together with global rotations and visual polarities (e.g., Howard and Childerson, 1994), and this can be one of the reasons roll vection measured in the current experiments tended to have a relatively longer latency and shorter duration. A future experiment may investigate roll vection by introducing the roll motion of the visual frame manipulated independently of the pattern rotation.

### Conflict of interest statement

The Guest Associate Editor Wataru Teramoto declares that, despite having collaborated on the Research Topic (The Future of Vection) with author Shinji Nakamura, the review process was handled objectively and no conflict of interest exists.

## Footnotes

^1^In this study, all participants executed experimental trials both for experiments 1 and 2 sequentially on different days; they observed the star stimulus employed in experiment 1 first, and then experienced the face stimulus in experiment 2. Although there were inter-experimental intervals long enough to avoid perceptual adaptation, one might still be skeptical that there was a kind of cognitive effects (e.g., habituation) and the difference of vection strength between the experiments could be partly explained by a fixed experimental order. This possibility would also be considered in future studies.
